# Eye Conformers as Socket Expanders in Children: Experience at a Tertiary Eye Hospital in Central Saudi Arabia

**DOI:** 10.7759/cureus.13465

**Published:** 2021-02-21

**Authors:** Nusrat Changal, Rajiv B Khandekar

**Affiliations:** 1 Oculoplasty/Anaplastology Clinic, King Khaled Eye Specialist Hospital, Riyadh, SAU; 2 Epidemiology and Public Health, King Khalid Eye Specialist Hospital, Riyadh, SAU; 3 Ophthalmology, Faculty of Medicine, University of British Columbia, Vancouver, CAN

**Keywords:** anophthalmia, childhood blindness, orbit, birth defect, conformers

## Abstract

Purpose

To share our experience with pediatric orbital expansion using eye conformers for anophthalmia and microphthalmia and parental feedback on outcomes.

Methods

Cases of congenital anophthalmia and severe microphthalmia were managed with eye conformers for orbital expansion and formation of lid fornices at the anaplastology clinic of King Khaled Eye Specialist Hospital, Saudi Arabia. Data were collected on the globe adaptation process and the perceived achievements by the parents at different follow-up visits. Parental feedback was collected on their acceptance of eye conformer use to address anophthalmia and microphthalmia.

Results

The anophthalmia/microphthalmia annual prevalence was 1.7 per 10,000 live births in Saudi Arabia. Of the 45 sockets treated for orbital expansion since 2014, 15 children were managed by using eye conformers. Six children had a bilateral birth defect. Severe microphthalmia was in seven children while eight children had anophthalmos. At the first visit, small eye conformers (nine), stem eye conformer (four), symblepharon ring (one), and hydrogel eye conformer (one) were fitted. After multiple visits and follow-ups, at the two-year follow-up, seven (46.7%) children were fitted while three (20%) were under the process of prosthesis fitting, as volume expansion was satisfactory. Parents of these children replied that they prefer this method over others and would recommend others to follow the same.

Conclusions

Orbital expansion and lid fornices formation by using an eye conformer is effective, easy, and acceptable to parents. It can be initiated in the early months of a child’s life.

## Introduction

Congenital anophthalmia and microphthalmia are rare diseases that cause anomalous orbitofacial growth and significant visual morbidity. Congenital anophthalmia is the complete absence of the eye due to malformation of the optic vesicle during early gestation [1]. Microphthalmia is the presence of a hypoplastic or rudimentary eye at birth [1]. The prevalence of congenital anophthalmia and microphthalmia ranges from 0.2 to 3.0 per 10,000 births [2].

The absence of a normal-sized globe is associated with orbital bone and soft tissue retardation and hemifacial microsomia. Computed tomography (CT) and magnetic resonance imaging (MRI) scans enable clinicians to rule out associated neurological, renal, cardiac, or other defects [3]. The management of severe microphthalmia or anophthalmia involves stimulating orbitofacial growth. Different techniques and materials have been used to expand the orbital volume, including acrylic conformers, spherical orbital implants, mucous grafts, dermis fat grafts, bone and muscle grafts, inflatable balloon devices, and hydrogel expanders [4].

However, the growth of eyelids and orbital bones also require stimulation either concurrently or soon after addressing orbital volume expansion. Hence, the rehabilitation process should begin soon after birth. Parents often decline surgical management because their children are very young. In cases where surgical management is refused, progressive expansion therapy with custom eye conformers followed by custom ocular prostheses is a reasonable option. Previous studies have reported that socket expansion with self-inflating expanders and custom-made eye conformers produce similar outcomes [5].

A conformer is a clear acrylic shell fitted to an anophthalmic socket or in the case of a microphthalmia socket before fitting an ocular prosthesis. It helps in the formation of fornices and helps expand the socket. In the case of congenital contracted sockets, we need to change conformer size every six weeks to two months for size to expand the socket gradually. It is also used after an enucleation an evisceration of the eyeball over the implant inside the socket to hold the shape of the eye socket and allow the eyelids to blink over the shell without rubbing the suture line. The conformer shell holds the shape ready for the artificial eye.

At the King Khaled Eye Specialist Hospital (KKESH), Saudi Arabia, three experienced technicians at the anaplastology clinic provide eye conformer sizing, fitting, and follow-up services for children with congenital anophthalmia and microphthalmia. There is a relative paucity of literature on the use and outcomes of conformers for these congenital conditions. In this study, we present the management outcomes of eye conformers for pediatric anophthalmia and severe microphthalmia in relation to increasing orbital volume, offering cosmetic outlook, the stability of the prosthesis, and parental satisfaction.

## Materials and methods

This one-armed retrospective cohort study was performed from January 2020 to June 2020. The Institutional Research Board approved this study (2031-R). Written consent was waived due to the retrospective data collection. However, parents of some children provided written consent to use photos for research publications and presentations while maintaining the anonymity of the children. This study adhered to the tenets of the Declaration of Helsinki.

Children with contracted sockets due to congenital anophthalmia or microphthalmia, who were managed with eye conformers at the anaplastology clinic at KKESH between 2014 and 2019 were included in this study. Children with less than one year of follow-up after eye conformer fitting were excluded from the study.

**Figure 1:**
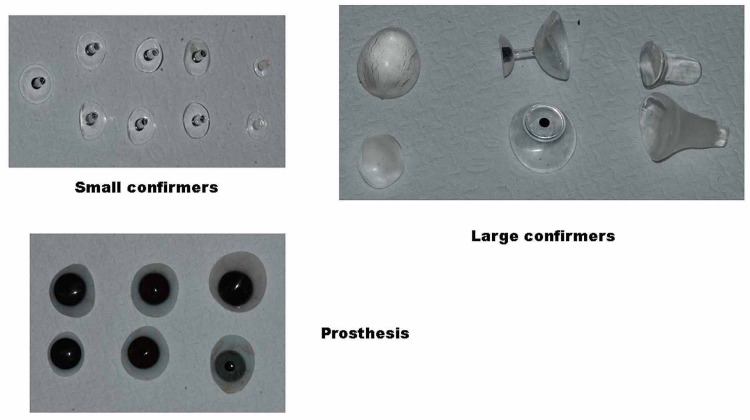
Small, medium, and large eye conformers and prosthetics that were used for orbital enlargement in anophthlamic and severe microphthalmic sockets

Electronic health records were used to collect data on patient demographics, the severity of the malformation, laterality (right or left eye), and other associated birth defects, including facial asymmetry. Data were also collected on the details of the first eye conformer and its status at follow-up visits every six to eight weeks in relation to extrusion, pain, crying, ulcer formation, and other signs and symptoms of adverse events. Different eye conformer sizes were used in the present study (Figure 1).

The fitting was retested every six to eight weeks, and a larger eye conformer was fitted based on the formation of the fornices, expansion of the orbit, and the loosening of the palpebral fissure.

At the two-year follow-up, a prosthesis was fitted into the orbit and measured. As we enrolled a pediatric patient population, MRI or CT was not performed for measuring orbital volume. Hence, in the current study, the description of the fitting process is based on the perception of the technician reviewing photographs at different stages of eye conformer fitting, replacement, and, finally, prosthesis implantation.

The change in orbital volume due to eye conformer fitting was defined as the difference in volume from pre-insertion to two years follow-up. Success was defined as no extrusion of the prosthesis, fornices that were deep enough, and a palpebral fissure of the affected side that matched the fellow normal eye in unilateral cases. A gradual increase in eye conformer size without any complications was also considered a success.

Parental feedback was collected on satisfaction with the facial aesthetics compared to the past and whether they would recommend this option to other parents.

The prevalence of anophthalmia and microphthalmia in relation to live births as reported in Saudi Arabia in 2019 was calculated [6]. The data were collected on a pretested form and transferred to the Statistical Package for the Social Studies for Analysis (SPSS 25, IBM Corp., Armonk, NY). Numbers and percentages are reported for categorical variables. Median and interquartile range (IQR) are reported for continuous variables.

## Results

The anaplastology clinic staff have assessed 513 sockets of 365 patients with anophthalmia or severe microphthalmia at the time of this study. The prevalence of anophthalmia was 1.7 per 10,000 live births. Since 2014, 45 sockets have undergone volume enhancement procedures, including 22 (48.9%) that received a polymethyl methacrylate (PMMA) implant, six (13.3%) that underwent an autologous dermis fat graft, two (4.4%) that received a polyethylene implant, and 15 (33.3%) received expanders.

The study cohort included 15 children with microphthalmia/anophthalmia treated with eye conformers for orbital expansion during the study period. The median age of the children on the day of the first visit was 375 days (IQR 190; 1170). Nine (56.3%) were boys and seven (43.7%) were girls. The youngest child was 48 days old. Six children had bilateral birth defects while nine children had unilateral anophthalmia. Severe microphthalmia was noted in seven children and eight children had anophthalmia.

**Figure 2:**
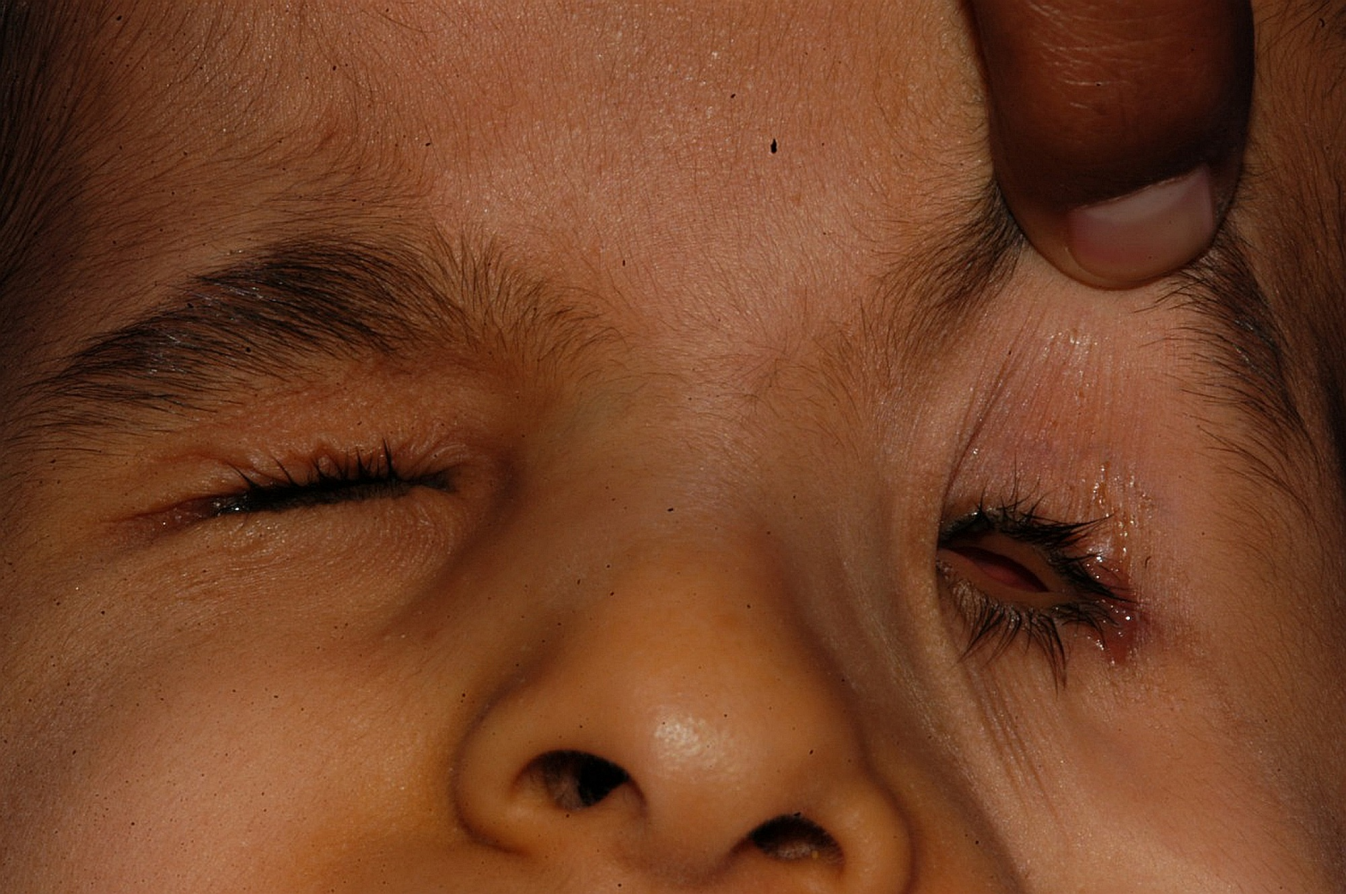
Bilateral anophthalmic orbital sockets at presentation Eyes are absent on both sides of the child's face, the palpebral fissure is very small, and the orbital contents are not visible even after pulling the left upper eyelid.

On the first visit, nine children were fitted with small eye conformers, of whom six received undersized eye conformers. Four children were fitted with stem eye conformers, and one child each received a symblepharon ring and hydrogel eye conformer. Figure 2 shows the status of the anophthalmic orbits of a child.

**Figure 3:**
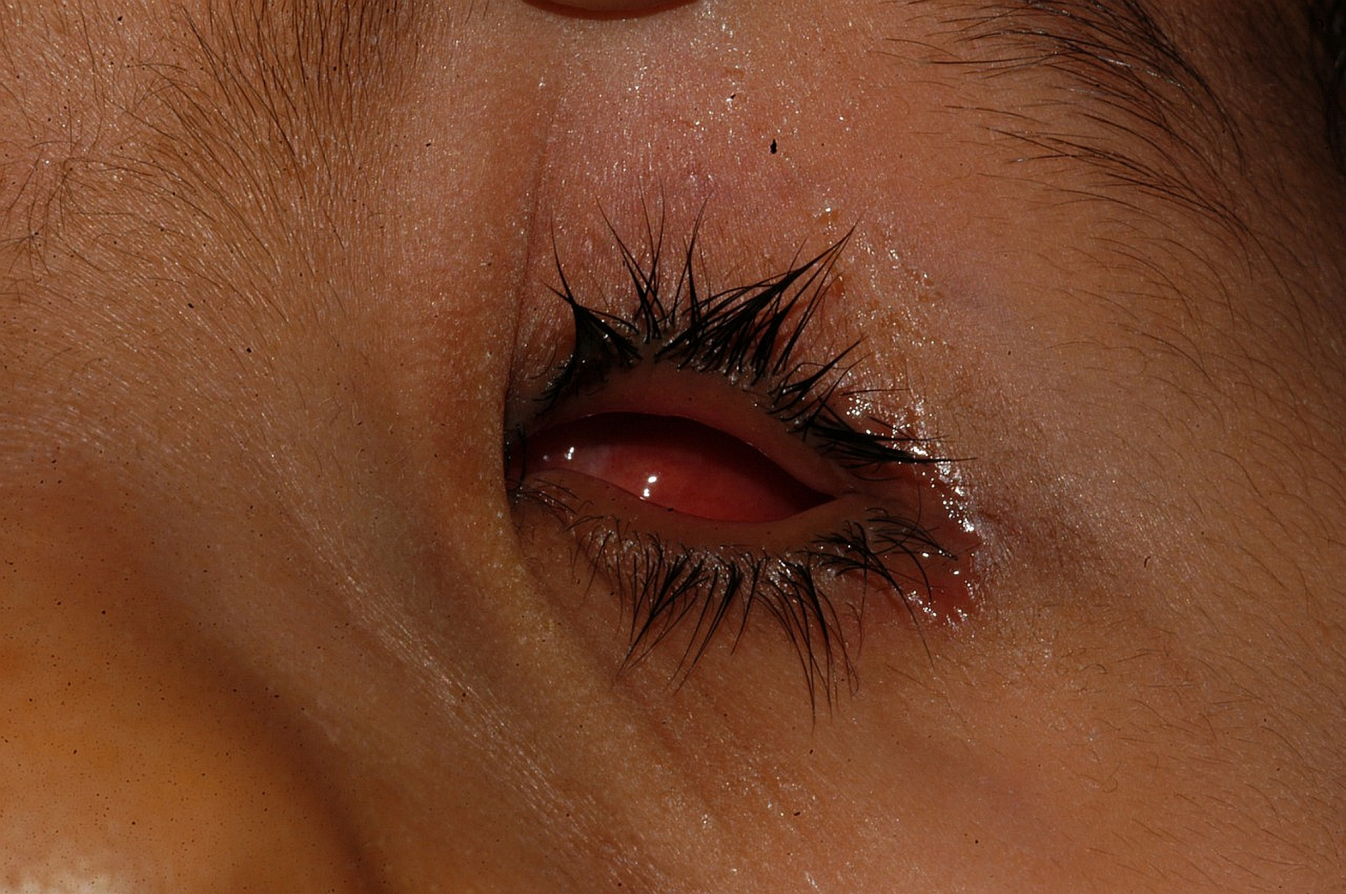
Small conformer fitted in the left orbit of a child with anophthalmia The child was fitted with a small conformer in the left orbit before six weeks. It is transparent and visible in the palpebral fissure.

On the second visit, six to eight weeks after the first visit, small eye conformers were continued for three children, six children were fitted with stem eye conformers, and two children each were fitted with medium and segmental eye conformers. One child who was fitted with a symblepharon ring type of eye conformer was more frequently monitored but the same treatment was continued. One child failed to present for the scheduled visit. Figure 3 shows a small eye conformer fitted in the right orbit of a child.

On the third visit at three to three point five (3.5) months after initial eye conformer fitting, one child continued with a small eye conformer, two had medium eye conformers, three had regional eye conformers, and six children had large stem eye conformers. One child’s fitting was ongoing while two children did not present for this follow-up visit.

**Figure 4:**
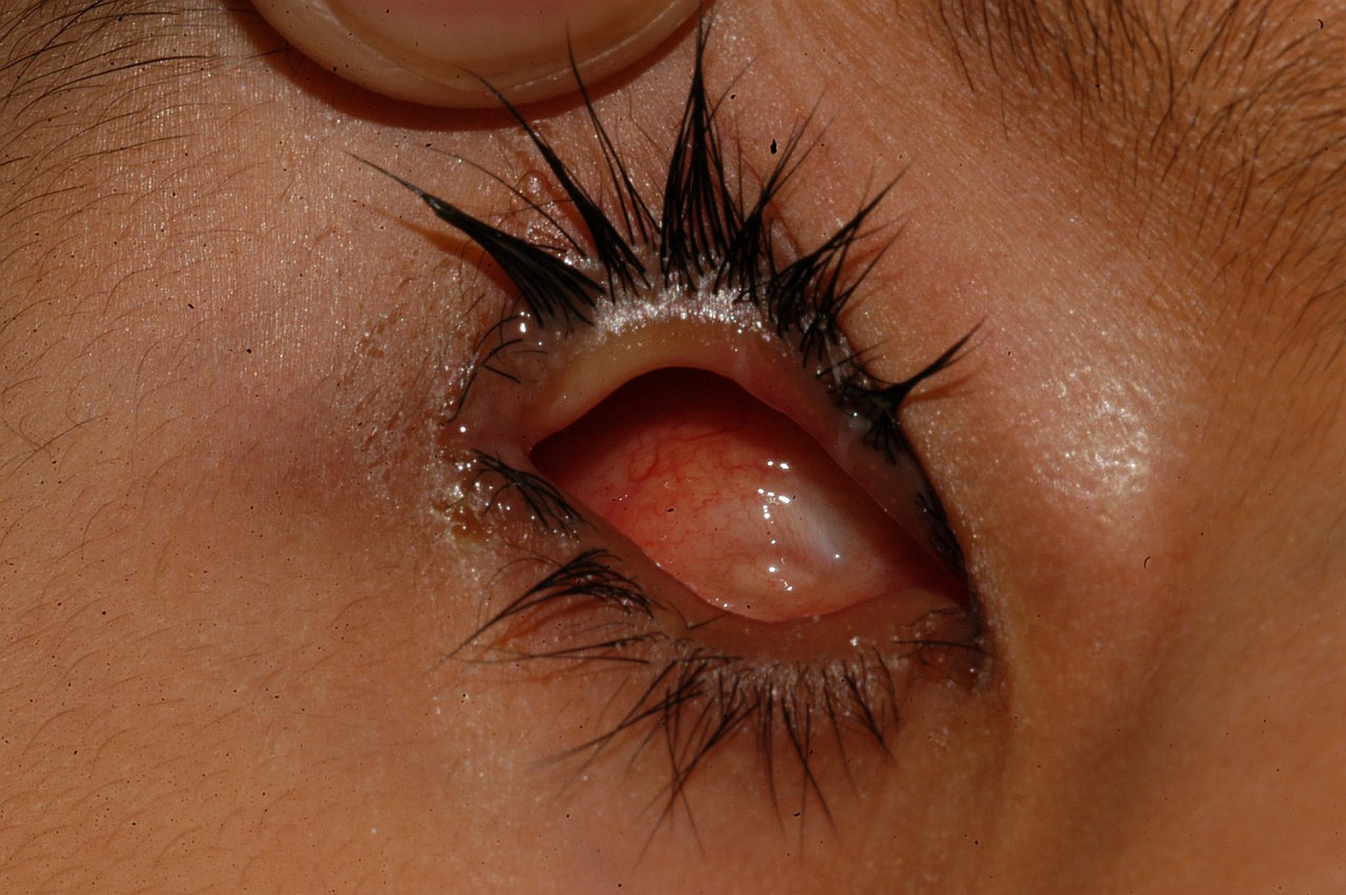
Right orbit and fornices of a child after one year of conformer fitting and replacing with a larger one gradually

On the fourth follow-up visit, one year after initial eye conformer fitting, five children were provided with a prosthesis, one with a regional eye conformer, and one with a large eye conformer. In three children, the fitting of the eye conformer was ongoing while three children were not included in the next step of treatment. Figure 4 shows the status of the orbit and fornices on the right side of the face of a child after one year of confirmer fitting and increase in size gradually.

**Figure 5:**
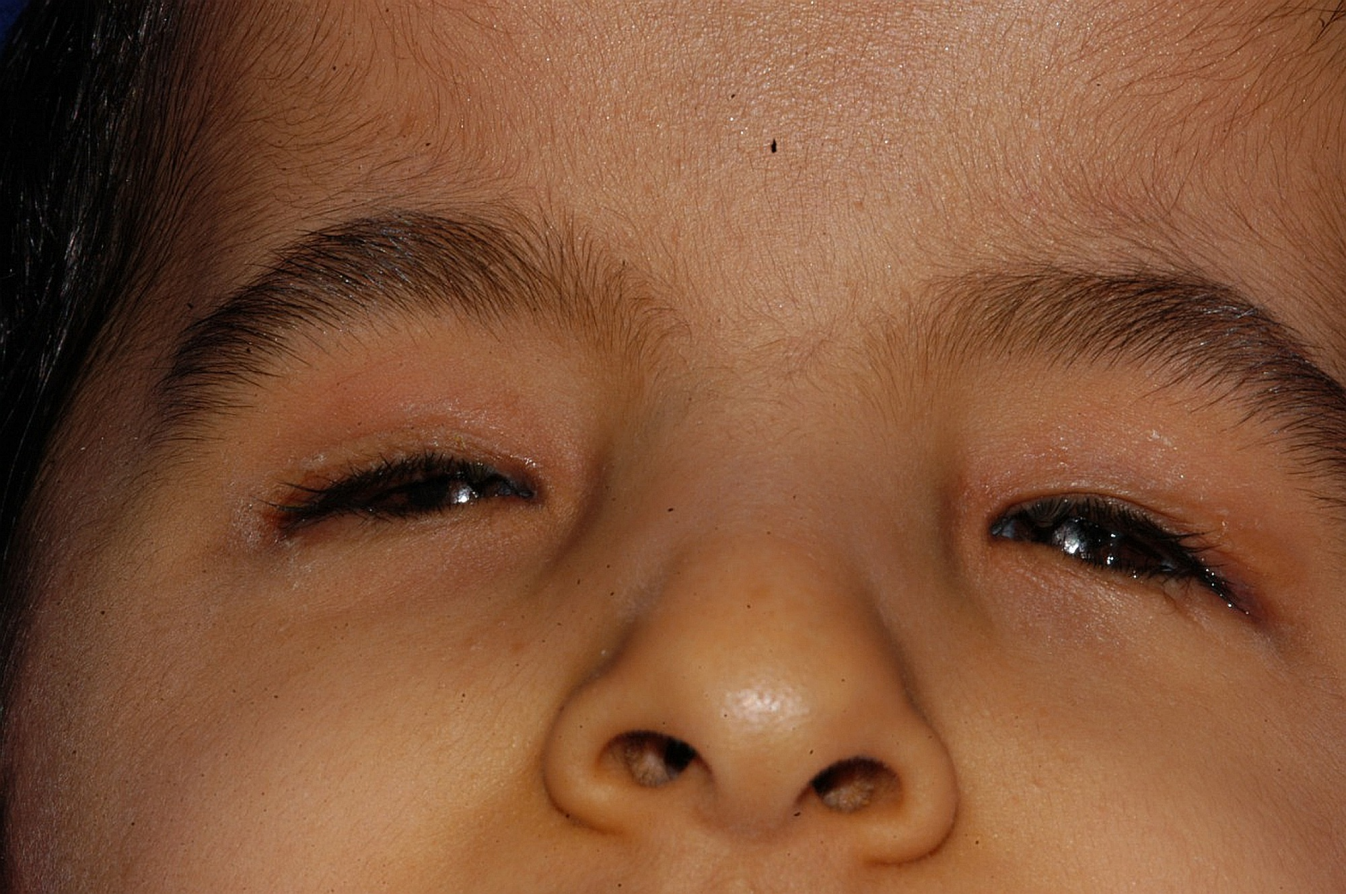
Child with a prosthesis in both orbits two years after enhancing the anophthalmic orbit with confirmers

At the fifth follow-up visit, two years after the initial conformer fitting, three more children were fitted with a prosthesis. One child’s prosthesis was increased in size. Figure 5 shows a prosthesis fitted in both orbits after it was expanded with the help of confirmers.

If we consider the loss to follow-up that could be due to many factors, including parents not satisfied with rehabilitation, or the child’s other ailments making them busy in other activities, the success rate of orbital expansion was 12/15 = 80%. At the time of writing, seven (46.7%) of 15 children were provided a prosthesis after volume expansion with conformers. Fitting of the conformer was ongoing in three (20%) children. The feedback from these three children’s parents suggested that they only prefer this method over surgeries for their children and would recommend this management to others prior to considering more invasive methods.

## Discussion

In this study, we present our experience with orbital expansion using a minimally invasive alternative that circumvented surgery on pediatric patients while achieving remarkable success. The procedure was also child-friendly and, therefore, more acceptable to the parents. This method can stimulate the development of the fornices and lids and, therefore, maintain the prosthesis.

The prevalence of anophthalmia and microphthalmia of 1.7 per 10,000 live births is lower than that reported in Texas, USA (3/10,000), and Denmark (3.6/10,000) [7-8]. Sixty percent of our cases were bilateral. In a published series of five children with microphthalmia, 40% had bilateral involvement [9]. The orbital and facial growth in the first year of life of a child without a globe results in significant facial asymmetry [10]. Irrespective of laterality, the diagnosis and management remain the same. Clauser et al have described the epidemiology, diagnosis, and management principles [11]. However, the use of orbital expanders are suggested after six months of age in a child with a disability. Morrow et al cautioned that if the management of the orbit does not commence in infancy, good results may not be achieved if interventions are initiated during childhood [12]. Our method of using conformers could commence soon after birth with parental consent. Hence, a thorough discussion of the benefits (and risks) of early intervention with the parents is fundamental to achieving good outcomes.

Although plastic surgeons currently recommend using hydrogel expanders, similar outcomes have been reported with eye conformers [13]. In our institute, we have noted complications of silicone implant in two cases of congenital anophthalmia [14]. This resulted in offering a safer and more acceptable alternative for expanding the orbit for parents who refuse surgery for their children. The different methods of orbital expansion of contracted sockets in children, along with their benefits and disadvantages, are described in Table 1.

**Table 1:** Comparison of different methods used to expand severe microphthalmic/anophthalmic orbital socket in children The method used to expand the orbit in the present study is compared to the other four methods showing benefits and weaknesses with the possible complications and limitations of each method. PMMA: polymethyl methacrylate

	Implant (Not integrated; PMMA, silicone or integrated = hydroxyapatite; polyethylene)	Dermis fat graft	Hydrogel expander	Silicone expander	External prosthesis
Indication	All types of anophthalmic socket	All types of anophthalmic socket	For very young kids with congenital anophthalmic socket	For very young kids with congenital anophthalmic socket	Anophthalmic socket grade 1 to 3, to be used over the anterior surface of the socket and with support on the fornix
Purpose	To replace orbital volume in enucleated or eviscerated eyes	To replace orbital volume in enucleated or eviscerated eyes	To enlarge orbital dimension in not developed orbits due to congenital problems	To enlarge orbital dimension in not developed orbits	To provide a better appearance
Surgery needed	Yes	Yes	Yes	Yes	No
Outcomes	Not integrated – no reaction between sphere and patient’s orbital tissues. Integrated – can have physical integration = polyethylene implant; or bio-integration = hydroxyapatite	Can suffer volume reduction due to graft absorption	If used in a young child need to be replaced for a large one Some types need to be empty externally (increased risk of infection)	If used in a very young child need to be replaced for a large one Some types need to be empty externally (increased risk of infection)	Good if there is fornix support
Complications	Not integrated: dehiscence, migration, extrusion Integrated: dehiscence, implant exposition, chronic inflammation	Dehiscence, necrosis (related to patient`s systemic conditions or bad surgical technique)	Dehiscence, migration, possibility of do not promote orbital enlargement resulting in asymmetry	Dehiscence, migration, possibility of do not promote orbital enlargement resulting in asymmetry	Can induce inflammatory reaction (papillary conjunctivitis)
Benefits	Replace volume in the socket with the external prosthesis, which can be thinner and not heavy	Replace volume in the socket. Can be replaced. Can be done with other procedures to enlarge the anterior surface of the socket	Favor the orbital development in young children	Favor the orbital development in young children	Can be replaced. Slow progress Less painful Accepted by parents due to cosmesis
Cosmetic effects	Good if associated with a well-adapted external prosthesis	Good when associated with a well-adapted external prosthesis	Good when associated with a well-adapted external prosthesis	Good when associated with a well-adapted external prosthesis	Good if the orbital volume and the fornix are suitable
Disadvantages	It is common that the need for a replacement integrated implant is expensive. All types of implants can suffer extrusion with time.	Need two surgical sites (the area to donate the graft and the socket)	Can be temporary and need replacement	Can be temporary and need replacement	Temporary and needs replacement. Need surgical procedures for orbital volume expansion

This study has some limitations, including the lack of objective measurements to document the orbital expansion during the study. However, eye conformer treatment began in the first year of life in most cases, and to mitigate needless exposure to radiation, we elected not to perform imaging studies (CT and MRI) on this pediatric population.

## Conclusions

Some proponents of orbital expansion and improving aesthetic outcomes for congenital anophthalmia and microphthalmia claim, with limited cases, that dermis fat grafts or hydrogel expanders are a better option. Each one has benefits and disadvantages. Multiple surgical interventions is not favored in children of tender age and parents also do not accept the risk of anesthesia for these procedures. But the procedure we have described to expand the orbit in children with anophthalmia with severely contracted sockets is a gradual procedure to expand, does not require surgery, is less painful to the child, and offers similar cosmetic results. It is less expensive and with minimum side effects of material being inserted in the orbital socket. Therefore, we described the procedure performed in our institute for others to understand and follow. We are not postulating that conformers are the best option but rather, they are an acceptable alternative to service providers and to parents due to the reversibility and perhaps the greater comfort for pediatric patients.
